# Occurrence of *bla_TEM_* and *bla_CTXM_* Genes and Biofilm-Forming Ability among Clinical Isolates of *Pseudomonas aeruginosa* and *Acinetobacter baumannii* in Yaoundé, Cameroon

**DOI:** 10.3390/microorganisms8050708

**Published:** 2020-05-11

**Authors:** Estelle Longla Madaha, Hortense Kamga Gonsu, Rhoda Nsen Bughe, Marie Christine Fonkoua, Collins Njie Ateba, Wilfred Fon Mbacham

**Affiliations:** 1 Biotechnology Centre, Faculty of Science, University of Yaoundé 1, Yaoundé, Cameroon; madahaestelle@gmail.com (E.L.M.); rhodabughe@yahoo.com (R.N.B.); 2 Laboratory of Bacteriology, Yaoundé University Teaching Hospital, Yaoundé, Cameroon; hgonsu@gmail.com; 3 Department of Disease, Epidemics and Pandemics Control, Ministry of Public Health, Yaoundé, Cameroon; 4 Bacteriology Service, Centre Pasteur du Cameroun, Yaoundé, Cameroon; mcfonkoua@yahoo.fr; 5 Antibiotic Resistance and Phage Biocontrol Research Group, Department of Microbiology, Faculty of Natural and Agricultural Sciences, North-West University, Mafikeng Campus, Private Bag X2046, Mmabatho 2735, South Africa; 6 Food Security and Safety Niche Area, Faculty of Natural and Agricultural Sciences, North-West University, Mafikeng Campus, Private Bag X2046, Mmabatho 2735, South Africa

**Keywords:** *Pseudomonas aeruginosa*, *Acinetobacter baumannii*, antimicrobial susceptibility testing, phenotyping, PCR, virulence and resistance genes

## Abstract

Background: *Pseudomonas aeruginosa* (PSA) and *Acinetobacter baumannii* (ACB) are non-fermentative bacteria mostly associated with nosocomial infections in humans. Objective: This study aimed to determine the antimicrobial resistance profiles and virulence gene of PSA and ACB previously isolated from humans in selected health facilities in Yaoundé, Cameroon. Methods: A total of 77 and 27 presumptive PSA and ACB isolates, respectively, were collected from the Yaoundé teaching hospital. These isolates were previously isolated from various samples including pus, blood and broncho-alveolar lavage. The identities of the isolates were determined through polymerase chain reaction (PCR) amplification of PSA and ACB specific sequences. Antimicrobial susceptibility testing (AST) was performed using the Kirby–Bauer disc diffusion method. Phenotypical expression of AmpC β-lactamases (*Amp*C), extended spectrum β-lactamases (ESBLs) and metallo β-Lactamases (MBLs) were determined using the combined disc method. Bacterial genomes were screened for the presence of β-lactamases *bla_TEM_* and *bla_CTXM_* genes using specific PCR. The pathogenicity of PSA and ACB was assessed through amplification of the *lasB, exoA, pslA* and *exoS* as well as *OmpA* and *csuE* virulence genes, respectively. Results: Of the 77 presumptive PSA isolates, a large proportion (75 to 97.4%) were positively identified. All (100%) of the presumptive 27 ACB harbored the ACB-specific *ITS* gene fragment by PCR. Twenty five percent of the PSA isolates produced ESBLs phenotypically while more than 90% of these isolates were positive for the *lasB, exoA, pslA* and *exoS* genes. A large proportion (88%) of the ACB isolates harboured the *OmpA* and *csuE* genes. *bla_TEM_* and *bla_CTXM_* were detected in 17 and 4% of PSA, respectively, while a much higher proportion (70 and 29%) of the ACB isolates possessed these resistance determinants respectively. Conclusion: Our findings reveal the occurrence of both virulence and drug-resistant determinants in clinical PSA and ACB isolates from patients in health care settings in Yaoundé, Cameroon, thus suggesting their role in the pathological conditions in patients.

## 1. Introduction

*Pseudomonas aeruginosa* (PSA) and *Acinetobacter baumannii* (ACB) are important causative agents of nosocomial infections in humans with more severe complications particularly in immunocompromised patients [1,2]. These pathogens are commonly isolated from wound infections, pneumonia and septicemia [3,4]. Infections due to PSA and ACB are difficult to eradicate because of their intrinsic resistance and their ability to acquire resistance against a variety of antimicrobial agents [5,6]. Generally, PSA and ACB display resistance to antibiotics using at least one of three different mechanisms [7]. These mechanisms include: decreasing the uptake of the drug and/or activation of efflux mechanisms to extrude the harmful molecule; structurally altering the targets for binding of the antibiotic and lastly by enzymatic or non-enzymatic modification or inactivation of the antibiotic, thus preventing them from interactions with their targets [7]. The potential to display resistance traits is worsened by the fact that some bacteria including PSA and ACB can use all three mechanisms to avoid destruction by antibiotics [7]. Based on reported evidence, β-lactam antibiotics are one of the most commonly used drugs worldwide [8,9], and this also applies to the treatment of infections caused by PSA and ACB [1,2]. The spread and persistence of β-lactam resistant and virulent bacteria strains in the environment most often results from the expanded use of this class of antibiotics [1]. Enzymes such as penicillinases, oxacillinases, cephalosporinases, and carbapenmases contribute most frequently towards the expression of β-lactam resistance phenotypes and genotypes among bacteria strains [8]. In addition, the potential to produce extracellular components such as exotoxin, phospholipase, alginase, elastase and biofilm forming determinants also enhance the pathogenicity of these bacteria [10,11]. Multi-drug resistant and virulence determinants, therefore contribute significantly to the severity of infections in their hosts [2]. To the best of our knowledge, there are few reports on the carriage, expression of resistance genes, and virulence determinants among bacteria isolates [12,13,14,15] especially PSA and ACB of clinical origin in Cameroon [16]. Moreover, it has been reported that, in Cameroon, resistance to commonly prescribed antibiotics is high [17] and this presents severe challenges to public health. This study was aimed at assessing the antimicrobial resistance and virulence determinants expressed by PSA and ACB isolates collected in some health settings in Yaoundé, Cameroon. Data generated may motivate policy makers to introduce a highly coordinated antimicrobial resistance monitoring program for the health care systems in Cameroon.

## 2. Materials and Methods 

### 2.1. Isolation and Handling of Bacterial Strains 

One hundred and four bacteria isolates comprising of 77 and 27 presumptive PSA and ACB from different clinical specimens, including pus, urine, sputum, bronco-alveolar lavage (BAL), sperm, high vaginal swab (HVS) and blood were collected from Yaoundé University Teaching Hospital (YUTH), Yaoundé Central Hospital (YCH) and Centre Pasteur du Cameroun (CPC) between January 2015 to March 2016. Presumptive identification of the isolates was performed in each collection site using either API 20 NE or VITEK 2. Bacteria isolates were revived on Tryptic Soy Agar (TSA) and transported to the Food and Drug Safety (FODRUS) Laboratory. The isolates were further identified using the catalase, oxidase, mannitol and citrate Simmons agar test. The Hajna Kliger media was used to assess lactose and glucose fermentation as well as the H_2_S production potentials of the isolates. All presumptive potential PSA and ACB isolates were inoculated in Brain Heart Infusion broth and stored at –20 °C. Isolates were later transported to the Antimicrobial Resistance and Phage Biocontrol Research Laboratory at the North West University, South Africa, for further analysis. 

### 2.2. Molecular Identification

#### 2.2.1. DNA Extraction 

Prior to identification, each isolate was cultured on nutrient agar and incubated aerobically at 37 °C for 24 h. Pure bacteria colonies were inoculated in 15 mL of Luria–Bertani (LB) broth (Merck, South Africa) and incubated aerobically at 37 °C for 24 h. Chromosomal DNA was extracted from exponential phase broth cultures using a DNA extraction kit (Zymo Research, CA, USA) according to the manufacturer’s instructions. Genomic DNA was quantified using a NanoDrop TM 1000 spectrophotometer (Thermo Fischer Scientific, Waltham, Massachusetts, MA, USA). 

#### 2.2.2. Polymerase Chain Reaction (PCR) Identification Tests

*Pseudomonas* species specific 16S ribosomal RNA and *P. aeruginosa* specific 16S ribosomal RNA gene sequences were amplified in all the isolates using oligonucleotide primer sequences that appear in Table 1. Amplification intergenic space sequences (*ITS*) specific to *Acinetobacter baumannii* was used to identify the isolates. Polymerase chain reactions (PCRs) were prepared as standard 25 μL volumes comprising 12.5 μL of 2X Master mix [0.05 U/μL *Taq* DNA polymerase, reaction buffer,4 mM MgCl_2_, 0.4 mM of each dATP, dCTP, dGTP and dTTp], 0.25 μL of each primer (1 μL), 1 μL template DNA (20–30 ng/ μL) and 11 μL nuclease free water.

All PCR reagents were obtained from Thermo Fisher Scientific, USA. The oligonucleotide primers for the different target sequences and amplification conditions are shown in Table 1. PCR was performed using a Thermal cycler (model C1000 Touch) supplied by BIO-RAD, Hercules, CA, USA. *E. coli* (ATCC 25922) was used as negative control strain in the study. Reference strains *P. aeruginosa* (ATCC 27853) and a previously confirmed and sequenced environmental *A. baumannii* isolate provided by the Antimicrobial Resistance and Phage Biocontrol Research Laboratory at the North West University, South Africa, were used as positive controls, respectively.

### 2.3. Antimicrobial Susceptibility Testing

Antimicrobial susceptibility testing (AST) of the isolates was determined by the Kirby–Bauer disc diffusion method according to a standard protocol [25]. Bacterial suspensions of 24 h cultures were prepared in 0.8% (*w/v*) normal saline solution to achieve the turbidity equivalent to 0.5 Mc McFarland standard. Aliquots (100 µL) of the bacterial suspensions were spread-plated on Mueller Hinton agar (MHA) plates and the following antibiotics piperacillin (30 µg^a^ and 100 µg^b^), piperacillin-tazobactam (30–6 µg^a^ and 100–10 µg^b^), ticarcillin (75 µg^a,b^), ticarcillin-clavulanic acid (75–10 µg^a,b^), cefepime (30 µg^a,b^), cefotaxime (30 µg^b^), ceftazidime (10 µg^a^ and 30 µg^b^), ceftriaxone (30 µg^b^), imipenem (10 µg^a,b^), meropenem (10 µg^a,b^), ciprofloxacin (5 µg^a,b^), levofloxacin (5 µg^a,b^), amikacin (30 µg^a,b^), gentamicin (10 µg^a,b^), netilmicin (10 µg^a,b^), tobramycin (10 µg^a,b^), doxycycline (30 µg^b^), minocycline (30 µg^b^), tetracycline (10 µg^b^) and trimethoprim-sulfamethoxazole (1–25 µg^a^ and 23–75 µg^b^) (Liofilchem s.r.l., Via Scozia, Italy) were placed on the inoculated plates. Antibiotics concentrations with superscripts “^a^” and “^b^” were used in the screening of PSA and ACB respectively. Plates were incubated aerobically at 37 °C for 24 h. Antibiotic inhibition zone diameter data (AIZD) of the different antibiotics were measured in mm and results were interpreted according to the recommendations of Comité de l’antibiogramme de la Société Française de Microbiologie/European Committee on Antimicrobial Susceptibility Testing (CASFM/EUCAST) 2016 [25]. TIBCO Statistica version 13.3 (StatSoft, TIBCO Software Inc., San Francisco, CA, USA) software was used to cluster organisms based on their AIZD data. Bacteria reference strains *E. coli* (ATCC 25922) as well as *P. aeruginosa* (ATCC 27853) and a previously confirmed and sequenced environmental *A. baumannii* isolate were used as negative and positive controls, respectively.

Furthermore, cluster analysis of antibiotic susceptibility data for PSA and ACB isolated in the study was determined using Wards algorithm and Euclidean distances on Statistica version 7.0 (Statsoft, Inc., Tulsa, OK, US).

### 2.4. Determination of Multidrug-Resistant (MDR) Patterns

Multidrug-resistant (MDR) isolates defined as resistance to at least three or more antimicrobial agents belonging to different classes was determined according to a standard protocol [26].

### 2.5. Phenotype Determination of Resistance Enzymes

The potential of PSA and ACB to produce enzymes hyperproduced cephalosporinase AmpC, extended spectrum β-lactamases, and metallo β-lactamases was determined by comparing the inhibition zone diameter data produced by the different antibiotics with and without a β-lactamases inhibitor.

#### 2.5.1. Detection of Hyper-Produced Cephalosporinase AmpC Phenotype

All isolates were routinely subjected to the phenotypic test for cephalosporinase AmpC using the cefoxitin-cloxacillin inhibition method as described by Tan et al. [27]. The test is based on the inhibitory effect of cloxacillin on the activity of the AmpC cephalosporinase. Cefoxitin (30 µg) discs supplemented with 200 µg of cloxacillin and only cefoxitin (30 µg) disc were used in the analysis. Isolates were considered AmpC producer if the antibiotic inhibition zone diameter (AIZD) produced by the cefoxitin + cloxacillin discs was higher by 4 mm when compared to those of the cefoxitin discs.

#### 2.5.2. Detection of Extended-Spectrum β-Lactamase Phenotype (ESBLs)

The combined disc method as recommended by Comité de l’antibiogramme de la Société Française de Microbiologie/European Committee on Antimicrobial Susceptibility Testing CA-SFM-EUCAST 2016 [25], was used to screen all isolates for the production of extended-spectrum β-lactamases.

(1) The Double Disc Synergy Test

Antibiotic discs ceftazidime, ceftriaxone, cefotaxime, cefepime and aztreonam each were placed about 30 mm around ticarcillin + clavulanic acid disc in pre-inoculated MHA plates. The plates were incubated aerobically at 37 °C for 24 h. The production of heart-shaped clear zones that distorts the zone of inhibition indicates positive results for ESBLs production [25].

(2) Combination Disc Method

Ceftazidime (30 μg) and clavulanic acid (10 μg) discs were placed separately on pre-inoculated MHA and plates were incubated aerobically at 37 °C for 24 h. Isolates were positive for the test if the AIZD produced by ceftazidime with clavulanic acid compared to that of ceftazidime alone is ≥5 mm [25].

#### 2.5.3. Detection of the Metallo-β-Lactamase (MBLs) Phenotypes

All isolates that were categorized as intermediate resistant (I) or resistant (R) to imipenem and/or meropenem were subjected to the MBLs phenotypic test using the imipenem-EDTA (ethylene diamine tetra-acetyl) inhibition method [28]. The principle of the test is based on the potential of EDTA inhibit metallo-β-lactamase activity. In order to perform the test, EDTA was prepared by adding 24.193 10^3^ μg of EDTA-2H_2_O disodium in 130.10^3^ μL of sterile distilled water so that 4 μL of the solution corresponds to 750 μg of 0.5 M EDTA per imipenem disc [28]. The pH of the EDTA solution was adjusted to 8 with NaOH solution. Bacterial suspensions were prepared in 0.8% (*w/v*) saline solution to obtain a turbidity equivalent to 0.5 McFarland’s standard and aliquots of 100 µL were inoculated on MHA plates by the spread-plate technique. Two imipenem (10 μg) discs were placed at 30 mm apart on the inoculated MHA plates. An aliquot of 4 μL EDTA solution corresponding to 750 μg of EDTA was added on one of the discs [28]. The inoculated plates were incubated aerobically at 37 °C for 24 h. The AIZD produced by imipenem discs (10 μg) alone and imipenem + EDTA (10 + 750) μg discs were measured and used to determine the potential of isolates to produce MBLs. For MBLs producers, the AIZD from imipenem + EDTA must be ≥7 mm that produced by imipenem alone.

### 2.6. Biofilm Formation Assay

All the 102 PAS and ACB isolates were selected based on their virulence gene and antibiotic resistance profiles and screened for their potential to form biofilms using a standard method [29,30]. Bacterial strains were grown aerobically at 37 °C for 24 h in tryptic soy broth (TSB) and later diluted 1:100 in TSB. Aliquots of 200 µL of the diluted broth cultures were transferred into the wells of 96-well polystyrene microtitre plates in triplicates. The plates were incubated at 25 °C and 37 °C for 24 h. Biofilm formation was performed at 25 °C because nosocomial (hospital acquired) infections caused by these bacteria have been associated with increased adherence to medical devices at room temperature [26]. In addition, the body temperature is 37 °C hence an important temperature range to assess the potential of these strains to adhere, survive, form biofilms and be able to subsequently cause disease in their hosts. TSB broth without bacteria cultures was used as a negative control. After incubation, plates were washed twice with PBS buffer to removed unattached cells and cells were stained with 200 µL of 1% (*w/v*) crystal violet for 1 h. Plates were washed five times with sterile distilled water to remove excess crystal violet stain, drained and air-dried. An aliquot of 200 µL of 95% (*v/v*) alcohol (>99%, Sigma-Aldrich) added to each well in order to dissolve crystal violet bound to biofilms. The optical density (OD_630 nm_) was measured using an enzyme-linked immunosorbent assay (ELISA) reader. The mean OD of each sample was compared to the optical density of the negative control, and the cut-off value (ODc) was calculated to be three-times the standard deviation of the blank OD mean plus the mean of the blank OD. Bacteria strains were classified as non-biofilm formers (ODs < ODc); weak biofilm formers (ODc < ODs < 2ODc); moderate biofilm formers (2ODc < ODs < 4ODc) and strong biofilm formers (ODs > 4ODc) [29,30].

### 2.7. Polymerase Chain Reaction (PCR)-Based Detection of Virulence and Resistance Determinants

The presence of PSA and ACB virulence determinants (*lasB*, *exoA*, *pslA* and *exoS*) and (*OmpA* and *csuE)* respectively were assessed by PCR assay with specific primers that appear in Table 1. The cycling conditions were based on previous protocols but with slight modifications [19,20,21,22]. β-lactam resistance genes *bla_TEM_* and *bla_CTXM_* were also amplified using previously described protocols [23,24]. PCR reactions were prepared as standard 25 μL volumes comprising 12.5 µL of 2X DreamTaq Green Master Mix, 0.25 µM of each primer, 1 µL of template DNA and RNase-nuclease free PCR water. Amplifications were performed using a Bio-Rad C1000 Touch^TM^ thermal cycler. All the PCR products were kept at 4 °C before they were separated by electrophoresis on 1% (*w/v*) agarose gel. PCR amplicons were visualised using a Chemi-Doc Imaging System (BIO-RAD ChemiDoc^MP^ Imaging System, Hercules, California, CA, USA).

### 2.8. Data Analysis

Excel 2016 software was used to organise and draw diagrams. TIBCO Statistica v13.3 (StatSoft, TIBCO software Inc., Tulsa, OK, USA) was used to build dendrograms using AIZD. Chi-square test was performed on SAS v. 9.4 to study the link between biofilm–formation ability of isolates and growth temperature 25 °C and 37 °C. Briefly, two variables were created using optical density obtained after biofilm formation assay carried out either at 37 °C or at 25 °C. A variable “temperature” refers to growth temperature and variable “biofilm” indicates the biofilm-formation ability. “Group A” was samples optical density (OD) obtained after incubation at 37 °C, and group B was samples optical density obtained after incubation at 25 °C. If there was biofilm formation, the value “1” was attributed and if there was no biofilm formation, value “0” was assigned. Each sample’s OD higher than blank OD was considered as a biofilm former. The hypotheses were as follows:

**Hypotheses (H0)**:  *There is independence between the two qualitative variables “group” and “biofilm”*

**Hypotheses (H1)**:  *There is a link between the two qualitative variables “group” and “biofilm”*

### 2.9. Ethical Approval

Ethical clearance was sought from the Central Region Ethical committee of the Central region, Cameroon, and an approval number (0259/CRESHC/2019) was assigned to the study.

## 3. Results

### 3.1. PCR Identification and Distribution of Isolates

A total of 104 bacteria strains comprising 77 and 27 presumptive PSA and ACB isolates were collected from the health facilities in Cameroon. The identities of a large proportion (75/77; 97.4%) of the PSA and all (27/27; 100%) of the ACB were positively confirmed by specific PCR as *Pseudomonas aeruginosa* and *Acinetobacter baumannii* respectively. PSA was frequently isolated in pus (33; 44%), urine (23; 30%), and broncho-alveolar-lavage (BAL) (13; 17%). On the contrary, ACB was more frequently detected in blood (16; 59%), urine (5; 18%) and pus (5; 18%). Table 2 outlines details of the frequency of the isolates from the different samples sources.

**Figure 1:**
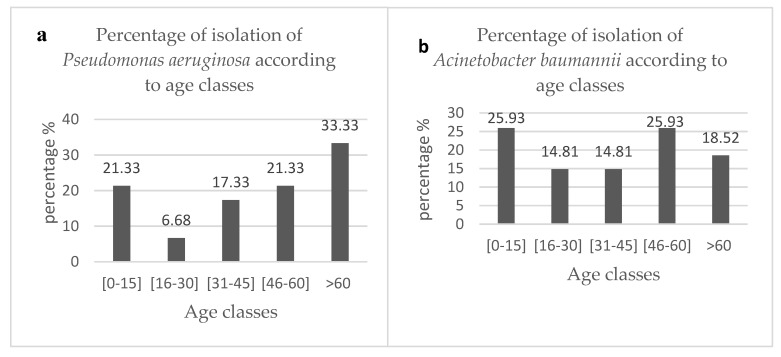
Percentage of bacteria isolation according to age classes. (**a**). *Pseudomonas aeruginosa;* (**b**). *Acinetobacter baumannii.*

The frequency of isolation of PSA and ACB from patients was also determined using age groups as variables. There was generally no significant difference in the proportion of isolates detected in patients from the different sexes. On the contrary, PSA and ACB were more frequently isolated in young (≤15 years old) and elderly (≥60 years old) individuals (Figure 1a,b).

### 3.2. Antibiotic Resistance Profiles

**Figure 2:**
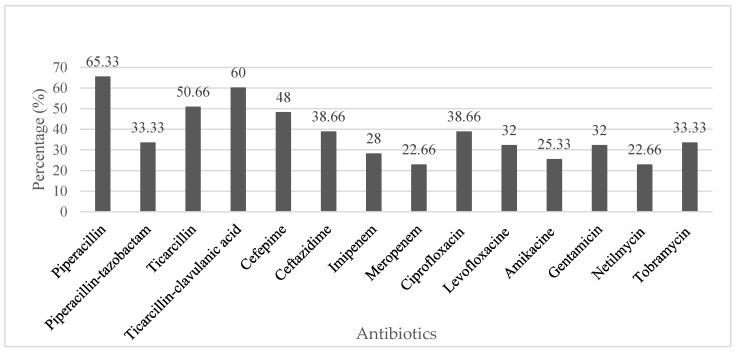
Antibiotic resistance of *Pseudomonas aeruginosa.*

**Figure 3:**
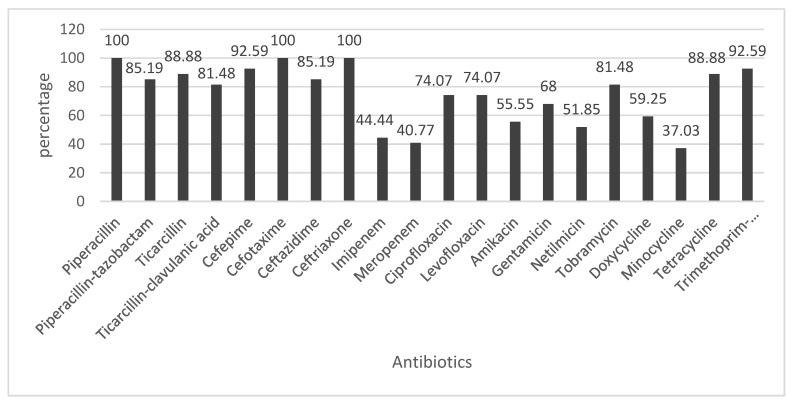
Antibiotic resistance of *Acinetobacter baumannii.*

In this study, approximately 50% of PSA isolates showed resistance to antibiotics belonging to the penam and cefem subfamilies. Resistance to carbapenems, fluoroquinolones and aminoglycosides was observed among 26%, 34% and 27% of the isolates, respectively. PSA showed the lowest level of resistance to meropenem 22%. The majority (13 of 20) of antibiotics used for AST of ACB recorded above 70% resistance frequency while minocycline showed the lowest resistance rate with a frequency of 46 %. It was observed that 40% of the isolates were resistant to carbapenem, more than 70 % were resistance to fluoroquinolone and aminoglycoside and about 60 % of resistance to tetracycline family. Figure 2 and Figure 3 present the percentages of PSA and ACB isolates antibiotics resistance respectively.

### 3.3. Resistance Enzymes

While 11 % of PSA produced the AmpC enzyme and none of the ACB was positive for AmpC production. Higher proportions (25 %) and (18%) of the PSA and ACB were positive for phenotypic production of ESBLs. ESBLs production was found only with the combined disc method, no positive result was found with the double disc synergy test (DDST). Despite that 6% of the PSA were positive for MBLs, none of the ACB produced these enzymes. A large proportion (66%) of PSA and all (100%) of the ACB displayed multidrug-resistance phenotypes.

### 3.4. Biofilm Formation Assay

**Figure 4:**
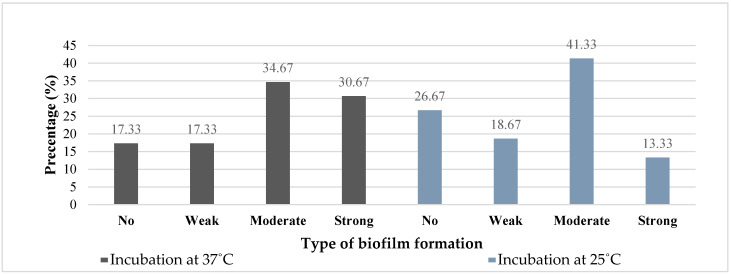
Percentage of *Pseudomonas aeruginosa* according to biofilm ability.

**Figure 5:**
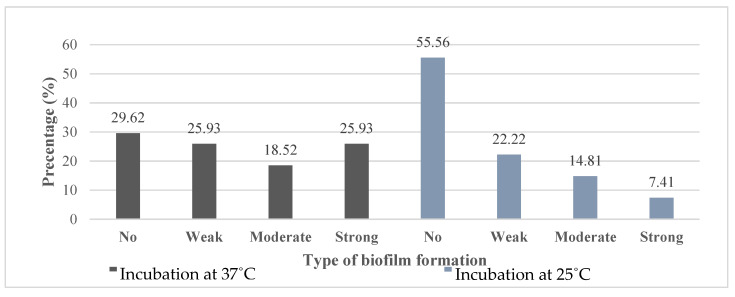
Percentage of *Acinetobacter* *baumannii* according to biofilm formation ability.

Large proportions (82%) of PSA and (70 %) of ACB were able to form biofilms at 37 °C and this ability was affected by a reduction in incubation temperature to 25 °C as the proportion of biofilm formers reduced especially for ACB (Figure 4 and Figure 5). Twenty-two percent of PSA lost their potentials to form biofilms when the incubation temperature was decreased to 25 °C from 37 °C while 13% of PSA also lost their biofilm forming capacity when the temperature was increased from 25 °C to 37 °C. Similar, fluctuations in biofilm forming potentials was observed for ACB at the different incubation temperatures (37 °C to 25 °C) and (25 °C to 37 °C). Irrespective of the incubation temperature, most of the PSA and ACB isolates were moderate and weak biofilm formers respectively.

Based on the Chi-square test, and the potential to form biofilms by ACB revealed that there was a direct relationship between biofilm formation and temperature (*p* = 0.0056) (Table 3). The probability for ACB to form biofilms at 25 °C and 37 °C was 27% and 65%, respectively. On the other hand, we found out that growth temperature of PSA did not affected biofilm formation (*p* = 0.0761).

### 3.5. Phenotypic Cluster Analysis of Isolates Based on Antibiotic Inhibition Zone Diameter (AIZD)

**Figure 6:**
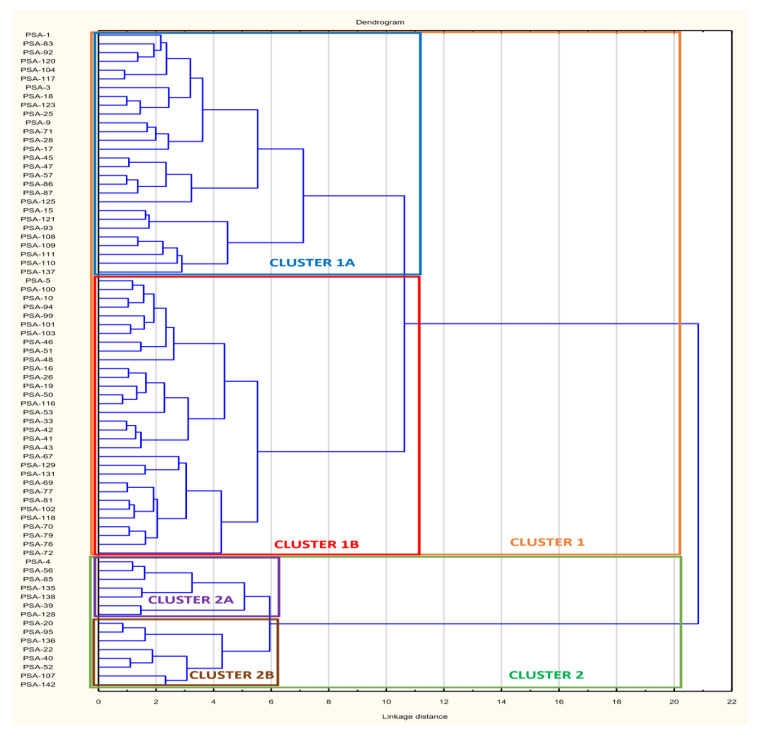
Relationship between PSA Isolates. Bacterial designations are based on sample origin and biofilm formation ability.

**Figure 7:**
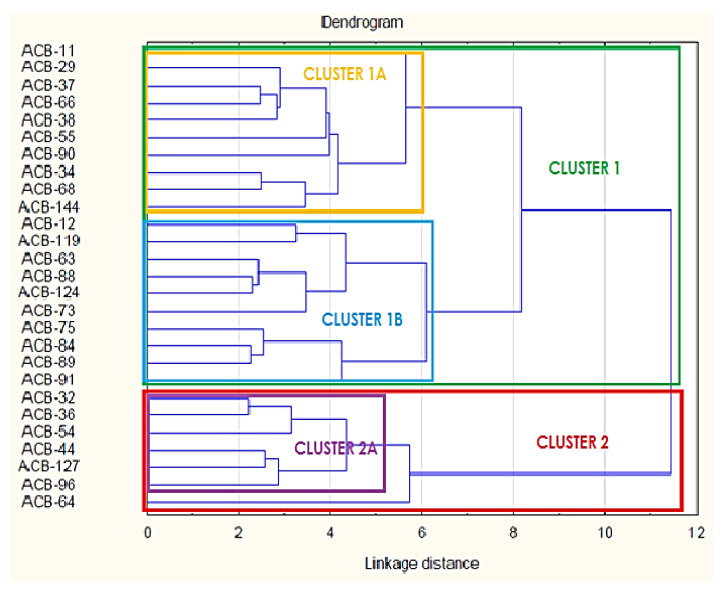
Relationship between ACB Isolates. Bacterial designations are based on sampling origin and biofilm formation ability.

In order to determine the phenotypic relationships based on antibiotic exposure histories and resolved the differences in AIZD of the multi-drug resistant PSA and ACB, isolates were subjected to cluster analysis. The dendrograms in Figure 6 and Figure 7 reveal 2 major clusters (1 and 2) for each species and each cluster is sub-divided in 2 sub-clusters (A and B) (Figure 6 and Figure 7). The clusters were further analysed for patterns of associations of isolates from the different sources and data is presented in Table 4. A large proportion (60; 80%) of PSA belong to cluster 1. Distribution of PSA on its dendrogram was matched with isolates source (Table 4), it was observed that a large proportion (25, 75%) of pus isolates, 11(84%) of BAL are found in cluster 1. In addition, a similarly large proportion (4, 80%) of the isolates from blood clustered in sub cluster 1A while 15 (65%) of the isolates from urine samples were present in cluster 1B. When the distribution of PSA isolates in the dendrogram was matched with results of their biofilm formation potentials (incubation done at 37 °C) (Table 4) it was revealed that a large proportion (53%) of non-biofilm formers were included in one sub-cluster (sub-cluster 1B). Given the great similarities in the isolates from the different samples, it may be important to mention that these public health facilities are close to each other and serve individuals from Yaoundé and its surrounding areas, it may suffice to suggest that the isolates had similar antibiotic exposure histories. However, information on the residence of the individuals from which the samples were obtained was not available.

### 3.6. Virulence Genes Identification

**Figure 8:**
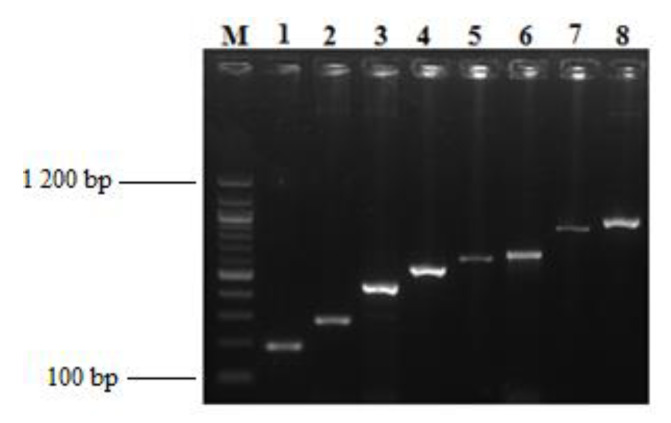
Gel electrophoresis of polymerase chain reaction (PCR) product of *Pseudomonas aeruginosa* identification, virulence and resistance genes amplified in this study. Each band represents the amplicon of one of the positive isolate for a given gene. **M**: 100 bp ladder: **1**: *exoA* (190); **2**: *lasB* (284 bp); **3**: *exoS* (444bp); **4**: *bla_CTX-M_* (550); **5**: *Pseudomonas* species; **6**: *psl* (656 bp); **7**: *bla_TEM_* (861bp); **8**: *Pseudomonas aeruginosa* specific gene (956 bp).

**Figure 9:**
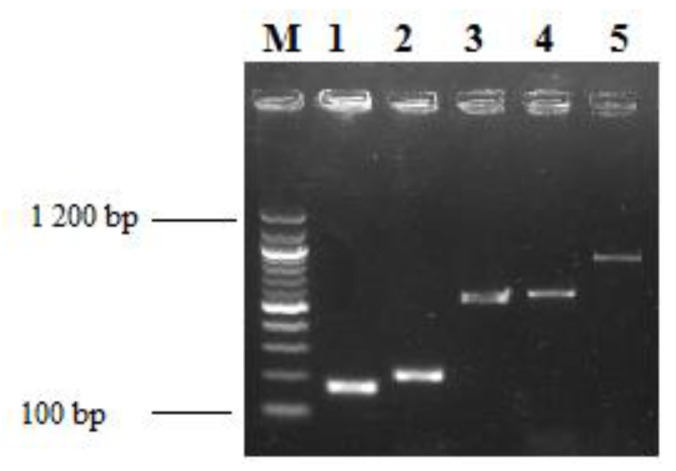
Gel electrophoresis of PCR product of *Acinetobacter baumannii* identification, virulence and resistance genes identified in this study. Each band represents the amplicon of one of the positive isolate for a given gene. **M**: 100 bp ladder; **1**: *csuE* (168 bp); **2**: *ITS* gene for *Acinetobacter baumannii* identification; **3**: *bla_CTX-M_* (550 bp); **4**: *OmpA* (578 bp); **5**: *bla_TEM_* (861 bp).

The *lasB*, *exoA*, *pslA* and *exoS* virulence genes were amplified in more than 90% of PSA. *OmpA* and *csuE* virulence genes were identify on 88% of ACB. Figure 8 and Figure 9 show the gel electrophoresis images of virulence genes and resistance genes amplified in PSA and ACB, respectively.

### 3.7. Resistance Genes Identification

Resistance genes *bla_TEM_* and *bla_CTX-M_* were respectively found among (13)17% and (3)4% PSA. Those genes were present among ACB with a proportion of (19)70% of *bla_TEM_* and (8)29% of *bla_CTXM_*.

## 4. Discussion

### 4.1. Bacteria Distribution

*Pseudomonas aeruginosa* and *Acinetobacter baumannii* are nowadays becoming important pathogens of severe public health significance especially due to their frequent involvement in health care-associated infections [31,32]. There have been incriminated in surgical wounds infections [31] and in pulmonary infections especially within nosocomial cases [32]. The main objective of this study was to confirm the identities of PSA and ACB associated with a variety of clinical complications in individuals who visited health care settings in Cameroon. PSA was most frequently isolated in pus (44%) and urine (30%) in this study and this corroborates with the results obtained in previous studies conducted in Cameroon and Ethiopia respectively [33,34]. On the contrary, a large proportion (59%) of ACB isolates from blood samples were positively confirmed as ACB and when compared to those from pus (18%) and urine (18%). This finding differs from those of Alemayehu et al. [34] and Alkasaby et al. [35] who isolated a large proportions of ACB in urine and endotracheal secretions. PSA and ACB are known to be opportunistic pathogens that should present severe complication in young children, individuals that are immune-compromised and the elderly. In this study, these pathogenic strains were dominant among patients within the age groups 0–15 years, 46–60 years and those who were older than 60 years of age. Given that patients belonging to these age groups are expected to be more susceptible to these pathogens due to a possibly weaker immune system, assessing the virulence and antibiotic resistance profiles of the isolates provides an indication of their pathogenicity.

### 4.2. Resistance Pattern

#### 4.2.1. Resistance to β-lactams

According to the World Health Organization (WHO) [36], there is a constant increase in antibiotic resistance worldwide and the emergence as well as spread of new resistance mechanisms is greatly hindering the ability to treat infectious diseases among susceptible patients globally. Drug-resistant bacterial infections are now regarded as a severe problem that significantly threatens public health thus requiring new approaches to combat them. In addition to the strategies put in place by WHO with the aim to prevent and control the spread of antibiotic resistance, a global action plan on antimicrobial resistance requires among others but most importantly to strengthen surveillance and research. This will improve our understanding of antimicrobial resistance and also create awareness within communities. Moreover, data generated from these investigations may significantly contribute in ensuring sustainable investments in strategies to counter antimicrobial resistance, optimize the use of antimicrobial agents thus reducing the development of infections in humans.

Despite the great therapeutic relevance of β-Lactam antibiotics, this group of drugs has been recently affected by the constant increase in bacteria resistance [37]. Previous studies have reported a steady increase in the spread of resistant PSA and ACB strains especially in hospital settings [38,39]. This report presents an assessment of the antimicrobial resistance profiles of these pathogens from clinical specimens in Cameroon with particular emphasis to drugs belonging to the sub-families penam and cephem. Our findings revealed that a large proportion (greater than 50%) of PSA were resistant to the penams (TIC, TCC and PIP) except for piperacillin-tazobactam (33%). Similarly, small proportions of these isolates from studies conducted in South Africa (19%) [40] and France (21%) [41] were resistant to piperacillin-tazobactam. Although the proportions (88%) of ACB from this study that were resistance to penam antibiotics (TIC, TCC, PIP and TZP) was slightly lower when compared to 89% resistance data reported by Alkasaby et al. [35], the detection of this high prevalence of resistant strains was a cause for concern.

PSA isolates in this study, displayed lower resistance (43%) to cephem antibiotics and this result is lower than previous studies conducted in Tanzania (63%) [42]. On the contrary, a much smaller proportion (13%) of isolates from Israel [43] were resistant to cephem. In this study, it was identified that 95% of the ACB isolates were resistant to cephem, and this was in accordance with the findings of a previous report in Egypt [35] and Tanzania [42] respectively.

Carbapenems are considered last resort antibiotics and recent baseline data reveals that as from the year 2006, resistance to carbapenems seems to be increasing in Cameroon [33,44]. Gangoue et al. [33] and Gonsu et al. [44] reported that only 6% and 8% of PSA isolates, respectively, of clinical origin in Yaoundé were resistance to carbapenems. In this study, a much larger proportion (24%) of PSA isolates were resistant to carbapenems. Our findings are similar to those reported in Spain, Italy, Latvia and Lithuania [45]. Based on available data, as at 2006, there was no evidence of the detection of carbapenems resistance among ACB isolates in Cameroon [33]. However in this study, up to 42% of the ACB isolates displayed resistance to carbapenems and these findings are similar to those reported in Spain and Hungary [45]. Contrary to our findings, data generated in Italy, South Korea and South Africa revealed that much higher proportions (80%–86%) of ACB were resistant to carbapenems [32,45,46]. Despite the fact that ACB isolates in this study were generally more resistant to the antibiotics when compared to PSA, data generated in a survey conducted in several countries including Cameroon revealed an increase in the accessibility and consumption of carbapenems and colistin [47]. Therefore, we suggest that this increased consumption of antibiotics has resulted to frequent exposure of bacteria to these drugs. The presence of increased antibiotic selective pressure during these years may have led to the development and dissemination of resistant strains at the expense of sensitive isolates.

#### 4.2.2. Resistance to Quinolones

Quinolone antibiotics are known to be the only antibiotics that are recommended for oral administration to patients suffering from *Pseudomonas aeruginosa* infections in many countries worldwide [48]. In the present study, only a small proportion (22%) of PSA isolates were most often resistant to quinolone antibiotics. Our findings are similar to those obtained in a study conducted in Israel (17%) but slightly lower than a report from Tanzania (35%) [42]. On the contrary, ACB isolates were most frequently (74%) resistant to quinolones. Despite the fact that the quinolone resistance profiles of the ACB isolates in this study were similar to those of a previous report in South Africa [46], but higher than that of a study in Tanzania (40%) [42] they were lower than an Egyptian data (92%). Given that *P. aeruginosa* isolates that are known to easily become resistant to quinolone antibiotics [48], thus severely limiting their effectiveness, the isolates from this study may present severe challenges to public health. 

#### 4.2.3. Resistance to Aminoglycosides

Resistance of PSA to different aminoglycoside drugs currently presents a serious threat to public health by limiting therapeutic options for treatment that are available. Moreover, the potential of *P. aeruginosa* to resist destruction by a variety of antimicrobial agents results from the frequent use of multiple drugs although at low dosages against the diseases caused by these strains [48]. To further assess the clinical significance of PSA and ACB from patients in Yaoundé, Cameroon, their resistance profiles against aminoglycosides was determined and our findings revealed that only 28% of the PSA were resistant to this drug. Moreover, a very small proportion (10%) of PSA previously isolated in Cameroon were resistant to aminoglycosides [44]. On the contrary, a very large proportion (70%) of ACB isolates in this study were resistant to aminoglycosides. This finding though high, remains lower than a report from Egypt (91%) [35]. Among four aminoglycosides tested in this study, PSA and ACB displayed high levels of resistance against tobramycin (33% and 81%, respectively) when compared to netilmicin (22% and 51%, respectively). These results are in agreement with those in previous reports [41,49]. Despite the fact that our isolates displayed similar levels of resistance to aminoglycosides when compared to these previous reports [41,49], the inherent abilities of PSA and ACB to form biofilms may enhance their potentials to resistant a variety of antibacterial agents especially when confronted with different environmental conditions [50]. These data may play a great role creating awareness on the need to adhere to the recommended dosage of antibiotics, let alone adherence to proper hygiene practices, especially in a country like Cameroon.

#### 4.2.4. Resistance to Tetracyclines

In the present study, a large proportion (89%) of ACB was resistant to tetracycline and this was similar to the findings of Meshkat et al. [51] who recorded 90% resistance against this drug. Although tigecycline was not used in this study, it was reported in a previous report that less than 2% of ACB strains were resistant to this antibiotic [30] and it is therefore suggested that the drug may be useful in the treatment of infections caused by MDR *Acinetobacter baumannii* [52,53].

#### 4.2.5. Resistance to Trimethoprim-Sulfamethoxazole

Baseline studies conducted in Cameroon some thirteen years ago revealed that only 30% of ACB isolates were resistant to trimethoprim-sulfamethoxazole [33]. However, data obtained in this study revealed a huge increase in the proportion of ACB (92%) that were resistance to trimethoprim-sulfamethoxazole. The very high resistance of the ACB isolates from this study against trimethoprim-sulfamethoxazole may be associated with the dissemination of resistant multi-drug resistant bacterial clones emerging from the expanded consumption of this antibiotic in Cameroon [9]. In general, recent data reveal a steady increase of both *Pseudomonas aeruginosa* and *Acinetobacter baumannii* towards most clinically relevant antimicrobial agents. This increase may be as a result of either poor infection control practices or misuse of antibiotics. Given that data previously reported in Cameroon that focuses on the practices and attitudes on the usage of antibiotics [54] revealed that up to 47% of clinically relevant antimicrobial agents can be accessed over the counter without any prescription, this provides opportunities for continued increase in bacteria resistance in the country. Due to this lack of strict control measures and the possibility of constantly detecting emerging multi-drug resistant strains, there is need to diversity search of possible alternative antibacterial agents with phage biological control assessments a valid option.

### 4.3. Detection of Antimicrobial Resistance Enzymes

Previous studies have reported a spread of resistant strains of PSA and ACB but with particular emphasis on the expression of enzymatic resistance markers [55,56,57]. The present study presents highlights or potentials of PSA and ACB to express the enzymes AmpC, ESBLs and MBLs.

#### 4.3.1. Phenotypic Detection of AmpC

From this study, none of the PSA isolates showed hyper-produced AmpC phenotype and this was contrary to the report of Gupta et al. [56] in which a large proportion of the isolates (50%) exhibited this phenotype. In India Gupta et al. [56] reported that only 11% of the ACB isolates were positive for the phenotype while Rynga et al. [55] reported a large proportion (99%) of AmpC producers. However, these differences could be attributed to the disparities in the protocols that were used in performing and interpreting the results. In the latter [56] report, AmpC producers were isolates that were resistant to cefoxitin. Therefore, this indicates the need for the standardisation and adherence to recommended laboratory protocols.

#### 4.3.2. Phenotypic Detection of Extended Spectrum β-lactamases (ESBLs)

ESBLs are serious threats to antibiotic therapy since they lead to bacteria resistance to all antibiotics belonging to the classes penicillin, cephalosporin and monobactam [58]. In this study, only 24% and 9% of PSA and ACB, respectively, displayed phenotypic expression of ESBLs. A report from Kauer et al. [59] in India revealed that 17% and 9% of PSA and ACB respectively were positive for the ESBLs phenotypes. On the contrary, two studies conducted in India revealed that a much higher proportions (38%) [55] and (12%) [60] of the isolates expressed ESBLs enzymes. Despite the fact that two different methods comprising DDST and the CDT were used in this study to screen for ESBL production, there was no positive result in the production of ESBL with the DDST while the antibiotics produced positive results for ESBL using the CDT. It can be deduced that combinatorial effects of antibiotics may be beneficial in treating nosocomial infections caused by either PSA or ACB. This observation corroborates with previous reports by Litake et al. [60] and Uddin et al. [61]. In order to treat infections caused by bacteria, antibiotics can be used either singly or in combination. However, with the ever-increasing bacterial resistance to currently used antimicrobial agents, the overall number of antibiotics that are effective against multi-drug resistant bacteria strains is declining. By convention, an approach to search for effective therapeutic options currently involves the use of antibiotics in combination, bacteriophage therapy, use of antimicrobial peptides, photodynamic therapy, antibacterial antibodies, phytochemicals and nanoparticles as antibacterial agents [62,63]. With this in mind, and based on the findings of this study it may suffice to indicate that the combinatorial effects of antibiotics may be beneficial in treating nosocomial infections caused by either PSA or ACB since combinatorial approaches involving two or more antibiotics or therapeutic agents may be effective in overcoming the individual limitations of single antibiotics [62,63].

#### 4.3.3. Phenotypic Detection of MBLs

In this study, none of the PSA isolates, were positive for the MBLs phenotype when compared to a small proportion (6%) of the ACB isolates that expressed the MBLs. Our findings contradict those of Gupta et al. [56] who obtained MBLs among 10% of the PSA isolates. Their finding as well as ours were lower than other reports previously documented in India and Pakistan in which 29% and 12% of PSA and ACB, respectively, were positive for MBL phenotypes [56,61]. In this study, large proportions (80% to 100%) of the isolates that expressed either AmpC, ESBLs or MBLs phenotypes were also MDR. This may be attributed to the possession of mobile genetic elements that most often harbour multiple genes encoding resistance to a wide variety of antibiotic belonging to different groups [64].

#### 4.3.4. Multidrug Resistance (MDR) Pattern

Multidrug resistance was detected among a large proportion (66%) of PSA. Despite the fact that our results for PSA was similar to those previously reported in India [45], it was higher than those of a study that was conducted in Italy (35%) [65]. In accordance with the finding that the resistance patterns of ACB reported worldwide reveal high levels of resistance to antibiotics belonging to almost all families, the detection of MDR among all (100%) of the ACB isolates of this study coincided with high levels of MDR strains reported in Iran (97%) [55], Italy (94%) [65] and India (91%) [66].

### 4.4. Biofilm Ability

Biofilm formation is one of the most virulence mechanism since the expression of this trait boosts survival of bacterial strains by enhancing their potential to adhere and resist antibacterial agents. In fact, it is known that 65% of all healthcare-associated infections (HCAI) originate from ability of bacterial contaminants to form biofilms [67]. In this study, 82% of PSA were biofilm formers at 37 °C and this is similar to reports presented in Iran and Serbia where more than 90% of the isolates formed biofilms [4,68]. This may be attributed to the inherent capacity for biofilm formation by PSA isolates thus providing them with the ability survive on abiotic environments, surfaces, hospital equipment and surgical instruments. Contrary to the findings of this study in which there was no significant relation between biofilm formation abilities of PSA isolates with variation (25 to 37 °C) in the growth or incubation temperature, it was previously reported that the biofilm formation potentials of PSA isolates differed with variation in growth temperature [69]. A defined assessment of the biofilm forming potentials of the PSA isolates using genetic markers revealed that a large proportion (100%) of PSA biofilm formers isolates harboured the biofilm gene *pslA*. Among the ACB isolates, a large proportion (70%) were biofilm formers at 37 °C, but this report was lower than those presented by Zeighanmi et al. in Iran and Yang et al. in Taiwan who found out that 100% and 93%, respectively, of the ACB isolates were biofilm formers [3,70]. The biofilm genes *OmpA* and *csuE* were detected in all the ACB biofilm formers and this finding corroborates with the report of Longo et al. [71]. In order to statistically assess the relationship between biofilm formation and temperature, using the Chi-square test, our findings revealed significant difference between biofilm forming abilities and culture temperature among ACB isolates. Similarly, significant variability had been reported among ACB isolated in Algeria [72]. Despite the similarities in the variation of biofilm-forming potentials with differences in temperature, our results contradicted theirs by having more biofilm formation by ACB at 37 °C rather than 25 °C while they obtained substantial biofilms at 30 °C than at 37 °C. Irrespective of the incubation temperature, large proportions the of PSA isolates were moderate biofilm formers, and this is in accordance with a previous report [3,68]. Moreover, ACB isolates were most frequently weak biofilm formers irrespective of the temperature and this observation was contrary to the findings of Zeighami et al. [3]. Given that the capability to form biofilms increases the survival of bacteria cells on dry surfaces [73] it is, therefore, important to increase hygiene practices in health settings in order to inhibit bacteria propagation and persistence in hospital environments. This will significantly control these pathogens and thus reduce the occurrence of both sporadic cases as well as outbreaks of nosocomial infections. This is motivated by the fact that biofilm forming nosocomial bacteria contaminants are frequently detected in various biomedical instruments that include catheters, implants, ventilators and dialysers thus worsening complications in the patients [74]. In our view, despite the new and highly stringent protocols that are being developed to curb this problem within health care settings, there is room for more improvements in order to reduce human suffering.

### 4.5. Cluster Distribution of the Isolates

Clustering based on inhibition zone diameters of PSA and ACB showed a great diversity in the antibiotic resistance profiles of the PSA and ACB isolates. The great similarities in the antibiotic resistant profiles of the *Pseudomonas aeruginosa* isolates obtained from pus and BAL that were present in cluster 1 together with 65% of the isolates from urine (found in cluster 1B) revealed similarities in antibiotic exposure histories. When the dendrograms were assessed for associations of isolates within clusters based on biofilm forming characteristics at 37 °C, there was a great similarity in which a large proportion (53%) of none biofilm forming *Pseudomonas aeruginosa* were present in the same sub-cluster (sub-cluster 1B). These findings reveal a very close association between similarities between biofilm forming potentials and antibiotic resistance among the PSA isolates of this study.

### 4.6. Virulence Genes Identification

A further objective of the study was to screen both PSA and ACB isolates for the presence of a variety of virulence genes. Although the *lasB* gene was frequently (98.6%) identified, more than 90% of PSA isolates harboured the *exoA*, *pslA* and *exoS*. This finding is higher to a previous report where they found 84 % PSA isolates bearing *lasB* gene [75]. The exotoxin A gene that is encoded by *exoA* plays a key role in cell death, a large proportion (90%) of the PSA isolates harboured this gene although, this was slightly lower than reports by Ahmed et al. [76] and Ruiz-Roldán [77] who recorded 100% and 98%, respectively. The *pslA* gene that is responsible for expression of the attachment organelle, cilia by PSA was detected in 92% of the isolates. The *exoS* gene that codes for a type-III cytotoxin involved in bacteria evasion and therefore facilitates an important stage in the pathogenesis of these strains, was harboured by 98% PSA isolates. Our findings are higher and different from those reported by Ruiz-Roldan et al., in Spain [77] and Ahmed et al., in Tunisia [76], who obtained a 65% and 77% respectively of the *exoS* gene. The frequent detection of virulence determinants among the PSA isolates in this study suggests very high pathogenicity especially when they harbour within highly vulnerable individuals. In addition, the detection of biofilm formation related genes *OmpA* and *csuE* in a large proportion of (88%) of *Acinetobacter baumannii* isolates coincides with the findings of Zeighami et al. in which 81% of the ACB isolates possessed the *OmpA* gene but contradicts a previous report in which all (100%) the ACB isolates were positive for the *csuE* gene [3].

### 4.7. Resistance Genes Identification

β-lactams are the most prescribed and consumed antibiotics in Cameroon [78] and the world at large. This has resulted in high levels of resistance against these drugs mainly due to the fact that the usage of β-lactam antibiotics is most often not limited to accessibility based on medical prescriptions. Several studies have highlighted the role of *bla*_TEM_ and *bla*_CTXM_ in *Pseudomonas aeruginosa* and *Acinetobacter baumannii* with respect to expression of β-lactams’ resistance [35,57,79]. In the current study, despite the fact that only 17% of PSA isolates harboured the *bla*_TEM_ gene, these data were higher than the findings of Abrar et al., in which none of the PSA isolates possessed the *bla_TEM_* gene [57]. The *bla_CTXM_* gene that is known to be the most widespread ESBL-resistant determinants among Gram-negative rod bacteria [35], was present in only a small proportion (4%) of the isolates screened in this study. Our data are contrary to those reported by Abrar et al., in Pakistan with 50% of PSA harbouring the *bla_CTXM_* gene. This very low prevalence of *bla_CTXM_* gene an ESBL gene in the PSA isolates of this study when compared to the phenotypic data (25%) strongly suggests that either others enzymes and/or determinants are involved in ESBLs activities or the *bla_TEM_* gene amplified within the current PSA isolates were in the majority an ESBLs variants. The *bla_TEM_* gene was detected in a large proportion (70%) of the isolates in this study and this is agrees the report of Alyamani et al. in Saudi Arabia [80]. With a large proportion (29%) of the *bla*_CTXM_ detected among the ACB from this study clearly contradicts a previous report in which only 1.8% of ACB possessed this gene [35].

## 5. Conclusions

The finding of this research has demonstrated high frequency of resistance among *Acinetobacter baumannii*. In fact, a relatively very large proportion (91%) of ACB was resistant to β–lactams (except carbapenem) and 100% were MDR. Phenotypic detection of resistance enzymes showed 25% of *Pseudomonas aeruginasa* isolates producing ESBLs. Virulence genes were amplified in more than 70% of both PSA and ACB. Biofilm formation assay achieved in this study highlighted the biofilm potential of these bacteria in our health settings and communities. β-lactamase genes *bla_TEM_* and *bla_CTXM_*, were present in our isolates with a particularly high proportion in *Acinetobacter baumannii* species. The present work has shown that PSA and ACB isolated from patients in Cameroon harbour a variety of virulence determinants and could contribute towards the pathogenicity of the isolates. The emergency of antimicrobial stewardship is more than ever crucial, as observed in the results of this study. Therefore, surveillance of resistance markers as well as virulence determinants among bacteria will help to control this superbug as seen in developed countries. Further studies will be conducted to determine their contribution in the dissemination of antibiotic-resistant genes among these pathogens. This would provide a clear picture of the health risks associated with the exposure of humans to these pathogens.

## Figures and Tables

**Table 1:** List of primers and polymerase chain reaction (PCR) conditions used in this study.

Target Gene	Primer Sequences (5’ to 3’)	Annealing Temperature	Amplicon Size bp	References
**Identification Genes**				
*Pseudomonas* spp.	F:GACGGGTGAGTAATGCCTA R:CACTGGTGTTCCTTCCTATA	53.6 °C	618	[18]
*Pseudomonas aeruginosa*	F:GGGGGATCTTCGGACCTCA R:TCCTTAGAGTGCCCACCCG	53.6 °C	956	[18]
***Pseudomonas aeruginosa* Virulence Genes**				
*lasB*	F:GGAATGAACGAAGCGTTCTCCGAC R:TGGCGTCGACG*AACA*CCTG	55 °C	284	[19]
*exoA*	F:TGCTGCACTACTCCATGGTC R:ATCGGTACCAGCCAGTTCAG	60 °C	190	[20]
*pslA*	F: TCCCTACCTCAGCAGCAAGC R: TGTTGTAGCCGTAGCGTTTCTG	60 °C	656	[20]
*exoS*	F:CGTCGTGTTCAAGCAGATGGTGCTG R: CCGAACCGCTTCACCAGGC	55 °C	444	[20]
***Acinetobacter baumannii* Identification Gene**				
*ITS*	F:CATTATCACGGTAATTAGTG R:AGAGCACTGTGCACTTAAG	55 °C	208	[21]
***Acinetobacter baumannii* Virulence Genes**				
*OmpA*	F:GTTAAAGGCGACGTAGACG R:CCAGTGTTATCTGTGTGACC	56 °C	578	[22]
*csuE*	F:CATCTTCTATTTCGGTCCC R:CGGTCTGAGCATTGGTAA	59 °C	168	[22]
**Resistance Genes**				
*bla_TEM_*	F:ATGAGTATTCAACATTTCCG R:TTACCAATGCTTAATCAGTGAG	50 °C	861	[23]
*bla_CTX M_*	F:CGCTTTGCGATGTGCAG R:ACCGCGATATCGTTGGT	56.9 °C	550	[24]

**Table 2:** Frequency of isolates according to specimen origin.

	Specimen Source	BAL	* HVS	Pus	Blood	Sperm	Urine	Total
*Pseudomonas aeruginosa*	Effectives	13	0	33	5	1	23	75
	Percentage	17.33	0	44	6.66	1.33	30.66	100
*Acinetobacter baumannii*	Effectives	0	1	5	16	0	5	27
	Percentage	0	3.7	18.51	59.25	0	18.51	100
Total	Effectives	13	1	38	21	1	28	102
	Percentage	12.75	0.98	37.25	20.59	0.98	27.45	100

* HVS: High Vaginal Swab, BAL: Broncho-alveolar lavage.

**Table 3:** Chi-square test result indicating the relationship of incubation temperature and biofilm formation for *Pseudomonas aeruginosa* (PSA) and *Acinetobacter baumannii* (ACB) isolates.

	ACB	PSA
*p*-value of Chi-square test	0.0056	0.0761

**Table 4:** PSA designations based on sampling origin and biofilm formation ability. The table was generated using data on the dendrogram.

**Cluster 1A** **N: 28(37%)**	Source of isolate * N (%)	Pus: 14(42) Blood: 4(80) BAL: 7(53)	**Cluster 2A** **N: 7(9%)**	Origin N (%)	Pus: 2(3) Blood: 0(0) Urine: 4(17) BAL: 1(7)
	Biofilm formation type at 37 °C	S: 9(39) M: 12 (46) W: 5 (38) No: 2(15)		Biofilm formation type at 37 °C	S: 4(17) M: 1 (3) W: 1(7) No: 1(7)
**Cluster 1B** **N: 32 (42%)**	Source of isolate N (%)	Pus: 11(33) Blood: 1(20) Urine: 15(65) BAL: 4(30) Sperm: 1(100)	**Cluster 2B** **N: 8(10%)**	Origin N: (%)	Pus: 6(18) Blood: 0(0) Urine: 1(4) BAL: 1(7)
	Biofilm formation type at 37 °C	S: 7(30.23) M: 11(42) W: 7(53) No: 7(53%)		Biofilm formation type at 37 °C	S: 3(11) M: 2(7) W: 0(0) No: 3(23)

* N: number; (%): occurrence percentage of the considered observation within the cluster; S: strong; M: moderate; W: weak; No: no biofilm formation ability.
